# Giant Ulcerative Basal Cell Carcinoma with Local Metastasis: A Case Report and Assessment of Surgical Techniques

**DOI:** 10.7759/cureus.6426

**Published:** 2019-12-20

**Authors:** Sahand Bamarni, Dereen Mohammed Saeed, Subhasis Misra, Darshan Thakkar

**Affiliations:** 1 Surgery, Brandon Regional Hospital, Brandon, USA; 2 Pathology, University of Illinois, Chicago, USA

**Keywords:** giant basal cell carcinoma, basal cell carcinoma, skin cancer

## Abstract

Giant basal cell carcinoma (GBCC) is a rare skin cancer characterized by an aggressive biological behavior with extensive local invasion, frequent metastasis, and associated poor prognosis. Wide local excision with sentinel lymph node biopsy is often warranted for this condition, and reconstruction by local rotational flap is one of the best surgical techniques for repairing similar skin cancers with a relatively large skin defect.

A 59-year-old man who was a former construction worker with a significant smoking history presented with a single giant suspicious chronic ulcerating skin lesion measuring 9 x 7 cm that proved to be a basal cell carcinoma (BCC) on his left shoulder. The patient was negative for enlarged or palpable lymph nodes and underwent a wide local excision and primary repair with a local flap. Despite negative margins, his follow-up visits at six, nine, and 10 months revealed numerous suspicious lesions that further required multiple local wide excisions that showed new basal cell carcinoma and recurrence to the left axilla. Given the invasiveness of his skin cancer, he was referred to oncology and later treated by chemoradiation.

Patients with multiple risk factors are associated with a higher incidence of more invasive skin cancer due to possible cumulative effects. The therapeutic approach for GBCC should involve multidisciplinary teams, with wide local resection of the tumor with possible sentinel lymph node biopsy, local rotational flap for reconstruction of the wide defect, and adjuvant chemoradiotherapy if necessary.

## Introduction

Basal cell carcinoma (BCC) is one of the most common malignancies in the US, with an estimated 4.3 million cases diagnosed annually [1]. BCC is a slow-growing, less aggressive tumor that rarely metastasizes. A rare and invasive subtype is a giant BCC (GBCC) that has more aggressive biological behavior with a tendency for deep tissue invasion and infiltration to extra-dermal tissue, as well as distant metastasis. GBCC is defined as a tumor larger than 5 cm in diameter that most commonly presents in the Caucasian population, with a male-to-female ratio of 2:1 [2]. Commonly affected sites are the face, back, and upper extremities. Pathologically, GBCC is often nodular or an infiltrative subtype of BCC, which can metastasize to regional lymph nodes or distant organs [2]. Patients with GBCC are at an advanced stage of BCC, usually due to a lack of timely medical intervention owing to patients’ negligence.

## Case presentation

**Figure 1:**
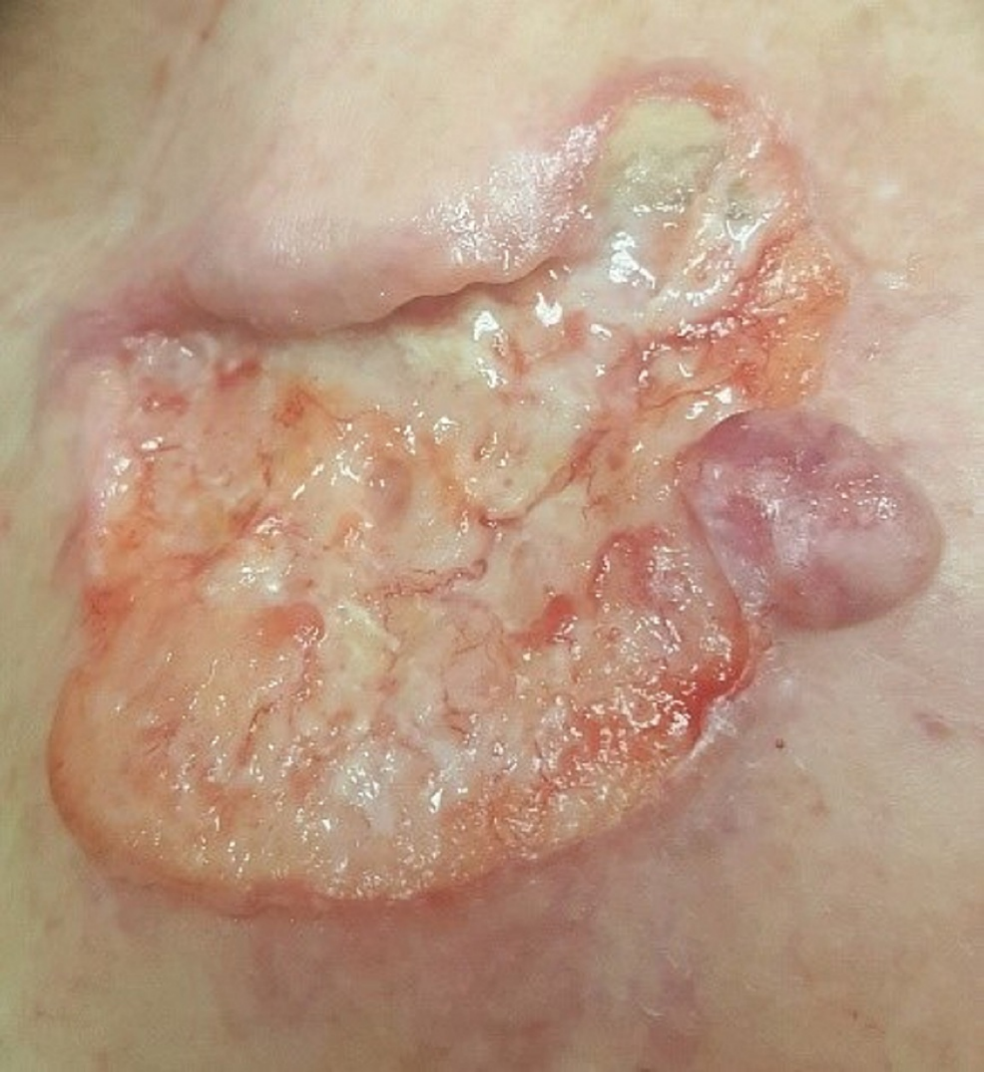
Large ulcerative lesion on the left shoulder with rolled edge on the medial side and nodular component on the lateral side

**Figure 2:**
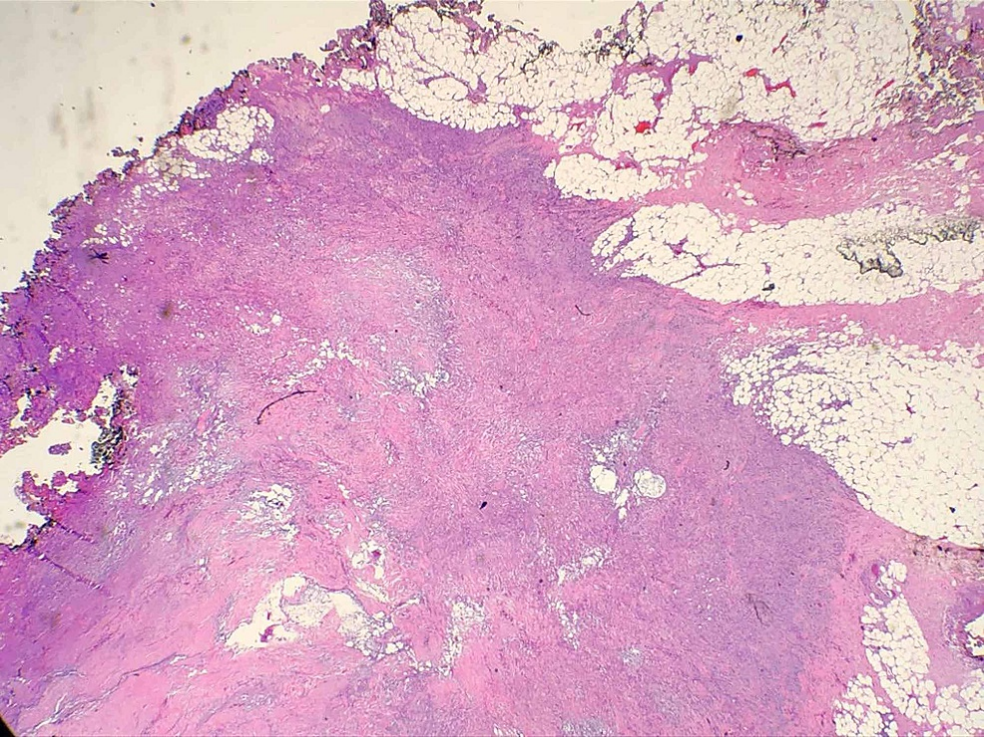
Histopathologic examination (H&E; original magnification: 100×) showing nests of basal cell carcinoma with the involvement of the margins H&E: hematoxylin and eosin

**Figure 3:**
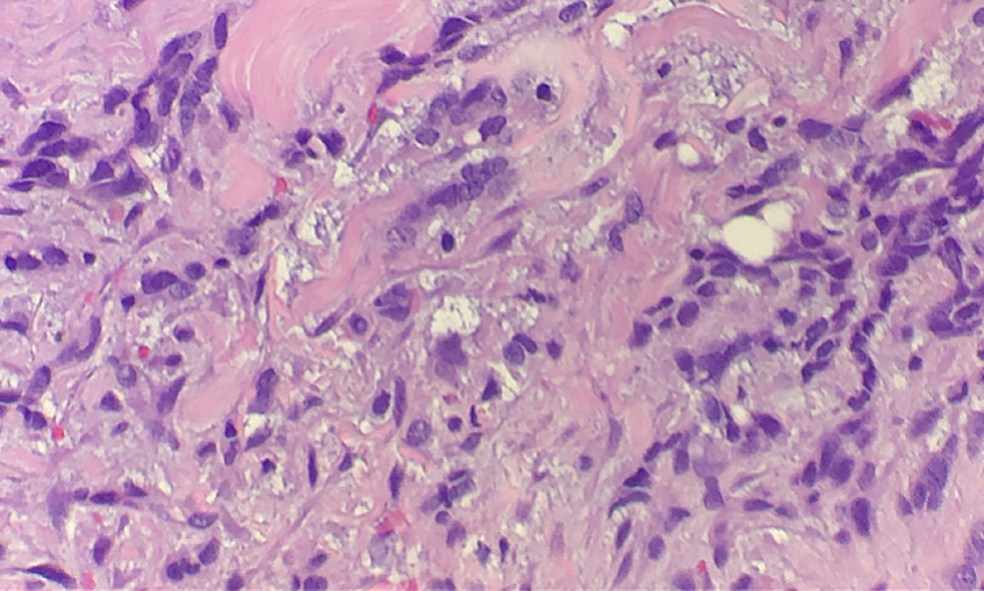
Histopathologic examination (H&E; original magnification: 400×) showing typical malignant cells of basal cell carcinoma H&E: hematoxylin and eosin

A 59-year-old white Floridian man, formerly a healthy construction worker with a smoking history of more than 30-pack-years, presented for an evaluation of a large (9 x 7 cm) chronic ulcerative lesion on his left shoulder (Figure 1). It had started as a small lesion and had progressively enlarged over several years. No lymph nodes were enlarged or palpable, and no other suspicious skin lesions were noted at the time of the initial evaluation. He underwent wide local excision and primary repair with a local flap that was complicated by flap rejection and necrosis. Local flap debridement was carried out, and negative pressure wound therapy was performed for six weeks with good outcomes. The final pathology demonstrated an infiltrating BCC of the morpheaform type with an extension to the subcutaneous tissue. However, all surgical margins were negative. At the six-month follow-up, another suspicious skin lesion on the left forearm proved to be BCC, and this was excised completely. At the nine-month follow-up, the patient presented with two new pink-colored superficial lesions on his right and left cheek, measuring 10 x 6 mm and 24 x 32 mm, respectively. Both of these were actinic keratoses and negative for BCC. At the 10-month follow-up from the initial excision, the patient presented with a new 3 x 4-cm left axillary mass. This lesion was removed with complete gross resection. The pathology result was conclusive for highly infiltrative BCC with positive margins (Figures 2 and 3). Given the invasiveness of his skin cancer, he was referred to oncology for targeted therapy.

## Discussion

BCC is typically a slow-growing, indolent skin cancer but may behave aggressively if neglected and left untreated for a long time [2]. GBCC is a rare type of BCC and associated significantly with high morbidity and mortality. A BCC larger than 5 cm in diameter is considered to be GBCC [2]. Based on TNM classification, GBCC would be characterized as a T3 BCC [3]. Archontaki et al. reviewed 51 reported cases of GBCC and evaluated the behavior of this type of skin cancer [2]. The majority of patients were Caucasian, with a mean age of more than 65 years. The most common primary sites were on the back followed by the face and then the upper extremities. The mean size of the mass was 15 cm, and the mean duration of the tumor presence was 15 years [2]. The majority of the GBCCs were nodular (55%) or infiltrative subtype (19%) and may progress to ulceration [2].

All GBCCs are considered high-risk BCCs, according to the National Comprehensive Cancer Network (NCCN) Clinical Practice Guidelines (Table 1) [4]. Although BCC rarely metastasizes, GBCCs have a greater tendency to metastasize, particularly in lesions larger than 10 cm [5]. Both local and systemic therapies are important parts of the treatment of GBCC due to the increased tendency of local invasion and the estimated metastatic risk of 17% [2].

Wide local excision of the lesion with histologically confirmed negative margins with a reconstruction of the defect followed by adjuvant chemoradiation gives a better outcome compared to radiotherapy or chemotherapy alone [6]. The NCCN recommends standard excision (SE) with more than 6-mm peripheral margins for high-risk BCC, while the European Dermatology Forum (EDF) and Cancer Council Australia, and Australian Cancer Network recommend SE using up to 10-mm peripheral margins [7].

The role of sentinel lymph node biopsy in BCC, in general, is not well established but is used for certain high-risk lesions such as those associated with lymph node enlargement or those demonstrating histologic evidence of lymphatic involvement [8]. Regional lymphadenectomy is recommended in cases of local lymphatic spread [2].

Medical therapy plays an important role in the management of locally advanced BCC or metastatic BCC. Vismodegib, the first US Food and Drug Administration (FDA)-approved drug for advanced BCC, provides a median overall survival of 33.4 months [9]. It is a hedgehog pathway inhibitor (HPI) and acts directly on the G protein [9]. Chemotherapy with cisplatin-based treatment is the most commonly used systemic regimen [10]. The clinical outcomes of chemotherapy vary from minimal response to complete remission. It should be reserved as a second-line treatment for those who fail to respond to HPIs due to the high toxicity and systemic side effects of cisplatin [10]. Other adjuvant medical therapies include topical imiquimod 5% cream. While approved by the FDA for the treatment of primary small superficial BCC, few reports have documented a successful treatment of GBCC with topical imiquimod 5% cream [11,12].

Recurrences at the primary site or other locations are considered one of the main challenges in GBCC management. A patient with BCC has a 45.2% risk of developing another BCC within 5 years [13]; therefore, lifelong or prolonged follow‐up with regular skin screening is recommended [14].

## Conclusions

Patients with multiple risk factors are associated with a higher incidence of more invasive skin cancer, possibly due to cumulative effects. The therapeutic approach for GBCC should involve a multidisciplinary team and treatment with wide local resection of the tumor, including a possible sentinel lymph node biopsy, local rotational flap for reconstruction of the wide defect, and adjuvant chemoradiotherapy if necessary.

## Appendices

**Table 1:** Low-risk vs high-risk BCC according to National Comprehensive Cancer Network Clinical Practice Guidelines on cutaneous BCC BCC: basal cell carcinoma

Tumor criteria	Low risk	High risk
Borders	Well-defined	Poorly defined
Site and size	Less than 20 mm in the trunk, extremities; less than 10 mm in head, neck, pretibia	Any size in the face, hands, feet; more than 20 mm in the trunk, extremities; more than 10 mm in head, neck, pretibia
Histologic subtype	Superficial nodular	Morpheaform, basosquamous, or micronodular
Local/systemic status	Previous normal skin, immunocompetent	Previous radiated site; immunocompromised
Invasion	No perineural invasion	Perineural invasion
Surgical excision	Negative margins	Positive margins
